# Early nutrition, growth and cognitive development of infants from birth to 2 years in Malaysia: a study protocol

**DOI:** 10.1186/s12887-016-0700-0

**Published:** 2016-09-29

**Authors:** Abdul Razak Nurliyana, Zalilah Mohd Shariff, Mohd Nasir Mohd Taib, Wan Ying Gan, Kit-Aun Tan

**Affiliations:** 1 Department of Nutrition and Dietetics, Faculty of Medicine and Health Sciences, Universiti Putra Malaysia, 43400 UPM Serdang, Selangor Malaysia; 2 Research Centre of Excellence, Nutrition and Non-Communicable Diseases, Faculty of Medicine and Health Sciences, Universiti Putra Malaysia, 43400 UPM Serdang, Selangor Malaysia; 3 Department of Psychiatry, Faculty of Medicine and Health Sciences, Universiti Putra Malaysia, 43400 UPM Serdang, Selangor Malaysia

**Keywords:** Early life nutrition, Growth, Cognitive development, Infants, Cohort study

## Abstract

**Background:**

The first 2 years of life is a critical period of rapid growth and brain development. During this period, nutrition and environmental factors play important roles in growth and cognitive development of a child. This report describes the study protocol of early nutrition, growth and cognitive development of infants from birth to 2 years of age.

**Methods/Design:**

This is a prospective cohort study of mothers and infants recruited from government health clinics in Seremban district in Negeri Sembilan, Malaysia. Infants are followed-up at 6, 12, 18 and 24 months of age. Pre-natal factors that include mother’s pre-pregnancy body mass index, gestational weight gain, blood glucose and blood pressure during pregnancy, infant’s gestational age, birth weight and head circumference at birth are obtained from patient card. Post-natal factors assessed at each follow-up are feeding practices, dietary intake, anthropometric measurements and cognitive development of infants. Iron status is assessed at 6 months, while infant temperament and home environment are assessed at 12 months. Maternal intelligence is assessed at 18 months.

**Discussion:**

Early life nutritional programming is of current interest as many longitudinal studies are actively being conducted in developed countries to investigate this concept. The concept however is relatively new in developing countries such as Malaysia. This study will provide useful information on early nutrition and infant development in the first two years of life which can be further followed up to identify factors that track into childhood and contribute to growth and cognitive deviations.

## Background

Nutrition in the early life has been shown to have a substantial influence on long-term health and development. There are critical periods when the system and organs of the human body are plastic and sensitive to the environment, and most of them occur in utero, except for the brain, liver and immune systems, which remain plastic after birth [1]. An insult or stimulus that occurs during a critical or sensitive period of development may have long-term effects on the tissue structure or functions, which is known as ‘programming’ [2]. Programming occurs as a result of adaptations for survival during the critical periods when the environment is compromised. For example, an undernourished mother during pregnancy will send a signal to her fetus that the environment outside might be harsh and the fetus will respond to this signal by reducing body size and altering metabolism for survival after birth [3]. Fetus will then reduce its insulin secretion and increase peripheral insulin resistance, directing more glucose to the vital organs, including the brain and the heart, and less to insulin-dependent tissues [4]. Peripheral insulin resistance and impaired pancreatic β-cell may result in glucose intolerance and hence diabetes when nutrition is abundance during adult life.

The concept of early life nutrition has been expanded to include milk-feeding period in the first 4 to 6 months of life and complementary feeding period up to the age of 2 years. Breastfeeding has been found to be associated with healthier body composition. Children who had been breastfed for at least 12 months were more likely to have lower fat mass at the age of 4 years than non-breastfed children [5]. Breast-fed infants were also reported to have lower risk of obesity, hypercholesterolemia, high blood pressures, cardiovascular diseases and type 2 diabetes in adulthood [2]. Early introduction of complementary foods (before 4 months of age) is associated with higher body mass index (BMI) in later childhood [6] and the risk of obesity was higher among formula-fed than breast-fed infants [7]. Flavor experience during this period could influence flavor acceptance and preference and subsequently food choice in later life [8, 9]. As breastfed infants received more variation of flavors from the foods that mothers consumed than formula-fed infants, the exposure to these flavors in the early life will encourage children to try new foods and enjoy a variety of nutritious foods in later childhood [9].

Nutrition plays an important role in pregnancy and infancy as these are the critical periods of brain formation that will serve as a foundation for the development of cognitive, motor and socio-emotional skills throughout life. Nutrient inadequacy during these periods may compromise the structural development of the brain [10]. For example, iron is an essential component of many enzymes that are involved in the oxidation-reduction reactions, synthesis and catabolism of neurotransmitters and production of myelin. Inadequate iron intake in the first 2 years of life, where the peak of myelinogenisis occurs, may have lasting negative effects on cognitive functions [11]. Infants with iron deficiency anemia (IDA) were more likely to have poorer cognitive development than non-anemic infants, and this cognitive impairment persisted even at 5 years old [12]. The effect of IDA on cognitive development could be mediated by poorer orientation or engagement and socioemotional functions associated with IDA [13].

Stunting, a common growth failure in young children, is mainly caused by poor nutrition and infections rather than genetic factors [14]. This growth failure is closely associated with poverty which restricts access to adequate quantity and quality food, clean water and sanitation, and quality primary health care. Poverty is also related to low maternal education, increased maternal stress and depression as well as inadequate cognitive stimulation at home. Both poverty and stunting are well-known risk factors for poor child development, including cognitive functions [15, 16]. However, factors such as child’s interaction with the environment, the timing and degree of nutrient deficiency and the possibility of recovery from undernutrition could determine the permanent effects of nutritional insults on cognition. Both nutrient supplementation and psychosocial stimulation are essential in improving cognitive development of undernourished children [17]. Stunted children who received a combination of nutritional supplements and early childhood development (ECD) intervention that include pre-school based activities and parenting education scored significantly higher in cognitive test than children who received only nutritional supplements for two years [18]. IDA infants receiving a combination of iron supplement and home intervention that includes support for mother-infant relationship were also found to score higher in cognitive assessment than infants receiving only iron supplement [19].

In Malaysia, many studies have examined factors associated with nutritional status and cognitive ability of children. However, given that early life nutrition can have an impact on growth and development of infants that may track into later childhood and adulthood, it is important to conduct a longitudinal study that look into this concept in Malaysia. This study will investigate the relationship between nutrition in the early life with growth and cognitive development of infants. Psychosocial factors such as home environment and infant temperament are also assessed, providing a comprehensive examination of factors that influence growth and development of infants in Malaysia.

### Aim of the study

This study aims to determine the association between early life nutrition with growth and cognitive development of infants in Malaysia. The specific research questions to be investigated in the study are:

1. What are the pre-natal and post-natal factors associated with growth and cognitive development of infants from birth to 2 years of age?
2. Is there any association between growth and cognitive development of infants from birth 2 years?

## Methods/Design

### Study design

This prospective cohort study follows mothers and infants for two years from three selected government health clinics in the district of Seremban in Negeri Sembilan, Malaysia. Infants are followed-up at 6, 12, 18 and 24 months of age. Ethical approval was obtained from Universiti Putra Malaysia Ethics Committee for Research Involving Human Subjects (JKEUPM), in accordance with the Declaration of Helsinki (FPSK(FR15)P012) and permission to conduct the study was obtained from the Ministry of Health Malaysia.

### Subject recruitment

Infants born between the year 2015 and 2016 are recruited at selected government health clinics during their first visit for immunization, usually between the ages of 0 to 2 months. Infants are screened for study eligibility criteria that include, male or female infant, infant less than 3 months old at recruitment, Malaysian, singleton and full-term (>37 weeks) infant, infant born with no physical or congenital abnormality and mother’s age more than 18 years at conception. Mothers are informed of the study and invited to participate in the study. Written consent is obtained from mothers prior to data collection. Subject recruitment has started in June 2015 and is currently on-going.

### Sample size

Sample size was calculated using a formula for cohort study with an adjustment for unequal groups [20]. Based on the ratio of the number of normal birth weight infants to low birth weight infants born in Seremban, Negeri Sembilan, which was 7:1 in 2006 [21] and the proportion of normal birth weight infants and low birth weight infants with low IQ in childhood [22], with 95 % power and 5 % significance level, a total of 491 infants are required for the study. Considering a possible attrition rate of 20 %, the number of infants to be recruited is 589 infants.

### Study measurements

#### General measurements

Socio-demographic background of parents and infants, which include parents’ age, ethnicity, educational attainment, occupation, household income, infant’s gestational age, date of birth and sex will be obtained through a questionnaire at recruitment. Table 1 shows the details of the types of data that will be collected at each follow-up.

**Table 1:** Data that will be collected at each follow-up of the study

Data	Recruitment	6 months	12 months	18 months	24 months
Socio-demographic background					
Parents’ age	●				
Parents’ ethnicity	●				
Parents’ educational attainment	●				
Parents’ occupation	●				
Monthly household income	●				
Infant’s date of birth	●				
Infant’s sex	●				
Infant’s gestational age	●				
Mothers					
Pre-pregnancy BMI		●			
Obstetrics history		●			
Anthropometric measurement during pregnancy		●			
Gestational weight gain		●			
Blood glucose during pregnancy		●			
Blood pressure during pregnancy		●			
Habitual food intakes in the third trimester		●			
Smoking and drinking behavior		●			
Maternal post-natal depression		●			
Maternal intelligence				●	
Infants					
Birth weight	●				
Length at birth	●				
Head circumference at birth	●				
Infant feeding practices		●	●	●	●
Complementary feeding		●	●	●	●
Energy and nutrient intake (24-h dietary recall)		●	●	●	●
Dietary diversity		●	●	●	●
Weight		●	●	●	●
Length/height		●	●	●	●
Head circumference		●	●	●	●
Waist circumference		●	●	●	●
Mid-upper arm circumference		●	●	●	●
Tricep skinfold thickness		●	●	●	●
Subscapular skinfold thickness		●	●	●	●
Iron status (infants)		●			
Infant temperament			●		
Home environment			●		
Cognitive development		●	●	●	●

#### Measurements of mothers

Information on mother’s pre-pregnancy BMI, obstetrics history, anthropometric measurements, gestational weight gain and biochemical data during pregnancy are obtained from patient card during the first follow-up at 6 month. Post-partum depression (within 3 months after birth) is assessed using a validated Malay version of the Edinburgh Postnatal Depression Scale (EPDS) [23, 24]. Mothers are asked to respond to 10 items on their feelings during the confinement period, usually within a month or two after delivery. Each item is scored on a scale of 0 (no risk of depression) to 3 (high risk of depression) with a maximum possible score of 30. Mothers are also interviewed on their smoking and drinking behaviors during pregnancy. During the follow-up at 6 months, mothers are requested to recall their habitual food intakes in the third trimester of pregnancy using a semi-quantitative food frequency questionnaire (S-FFQ), adapted from the Malaysian Adult Nutrition Survey 2003 [25]. Maternal intelligence is assessed during the follow-up at 18 months using Raven’s Standard Progressive Matrices (SPM) [26].

#### Measurements of infants

##### Anthropometric measurements

Infant’s birth weight, length and head circumference at birth are obtained from patient card at recruitment. Infants are measured for weight, length, head circumference, waist circumference, mid-upper arm circumference (MUAC), tricep and subscapular skinfolds (TSF and SSF) at 6, 12, 18 and 24 months. All measurements are conducted twice and recorded to the nearest 0.1 kg for weight, 0.1 cm for length/height, head, waist and mid-upper arm circumference and 0.1 mm for skinfold thickness. Weight-for-age, length-for-age, BMI-for-age, head circumference-for-age, MUAC-for-age, TSF-for-age and SSF-for-age are determined using WHO Anthro version 3.2.2 software [27]. Nutritional status of infants is then categorized based on the Z-scores using the WHO Child Growth Standards [28, 29].

##### Infant feeding practices

Infant feeding practices are assessed at 6, 12, 18 and 24 months. A questionnaire was developed to include all the 8 core indicators of infant and young child feeding (IYCF), which are breastfeeding initiation, exclusive breastfeeding, continued breastfeeding, introduction of complementary foods, dietary diversity, meal frequency, minimum acceptable diet and consumption of iron-rich fortified foods [30, 31].

##### Energy and nutrient intakes

Mothers are interviewed for infants’ dietary intake using a 24-h dietary recall at each visit at 6, 12, 18 and 24 months. Dietary data are analyzed using NutritionistPro^TM^ software (Axxya Systems, 2008) for energy and nutrients based on the United States Department of Agriculture (USDA) food database. Energy and nutrient adequacy is determined based on the Recommended Nutrient Intake (RNI) for Malaysians [32].

##### Dietary diversity

Dietary diversity is assessed at the age of 6, 12, 18 and 24 months using the Food and Agriculture Organization of the United Nation (FAO) dietary diversity questionnaire [33]. This questionnaire assesses intake of 16 food groups in the past 24-h as well as in the past week. A dietary diversity score is calculated based on the IYCF criteria, which includes intake of 7 food groups (grains, roots and tubers, legumes and nuts, dairy products, flesh foods, eggs, vitamin A rich fruits and vegetables and other fruits and vegetables). A score of 1 is given if the infants consume any food in each of the food groups. A score of 4 or more indicates a high likelihood that the infants consume foods from at least 1 animal source and 1 fruit or vegetable in addition to staple food [30, 31].

##### Iron status

Capillary blood sample of the infants is obtained through heel prick to assess hemoglobin concentration at 6 months [34]. HemoCue (Hb 201+) hemoglobin analyzer will be used to analyze hemoglobin concentration. A cut-off point of 11.0 g/dl hemoglobin concentration will be used to define a possible risk of iron deficiency anemia [35].

##### Infant temperament

Infant temperament is assessed using Revised Infant Behavior Questionnaire (IBQ-R) [36] at 12 months. The IBQ-R is a parent-report measure of infant temperament. It consists of 91 items with 14 subscales, including activity level, distress to limitations, high pleasure, low pleasure, soothability, falling reactivity, cuddliness, perceptual sensitivity, sadness, approach and vocal reactivity. Mothers are requested to report their infants’ behaviors during specific events in the past week using a 7-point scale, ranging from 1 (never) to 7 (always). If an event does not occur in the past week, then the mother can choose ‘does not apply’ option. Three broad dimensions of temperament, which are surgency/extraversion, negative affectivity and orienting/regulation will be obtained by summing the scores of the subscales. A higher dimension score indicates higher tendency towards the dimension.

##### Home environment

Home environment is evaluated using Infant Toddler HOME Inventory (IT-HOME) [37] at 12 months. The IT-HOME consists of 45 items with 6 subscales, including responsivity, acceptance, organization, learning materials, involvement and variety. Each items is given a score of 1 for ‘yes’ and 0 for ‘no’ based on observation or interview. A higher total score indicates higher quality of home environment. Parent-child interaction is also assessed in the IT-HOME through observation in the subscale for responsivity.

##### Cognitive development

Cognitive development of infants is assessed using Bayley Scales of Infant and Toddler Development, Third Edition (BSID-III) [38] at 6, 12, 18 and 24 months. The BSID-III measures development of infants and toddlers from 1 to 42 months of age across cognitive, language, motor, social-emotional and adaptive domain. In this study, only the cognitive domain is being measured. This includes assessment of sensorimotor development, exploration and manipulation, object relatedness, concept formation, memory and other aspects of cognitive processing [38]. The test is administered according to the infant’s age-specific start point. Each correct response is given a score of 1 and the total raw score is then converted into its composite score.

### Statistical analysis

Data will be analyzed using SPSS software version 23. Univariate and bivariate analyses will be used to describe the data. Logistic regression will be used to determine the relative risk of poor growth and cognitive development. Linear mixed model will be used to determine the effect of time on the relationship between pre-natal and post-natal factors with growth and cognitive development from 6 to 24 months.

## Discussion

Epidemiological studies support the relationship between early life nutrition and risk of adult diseases such as cardiovascular disease, diabetes mellitus and cancer. Fetal programming and developmental plasticity are plausible biological mechanisms that could explain the link between early life nutrition and adult diseases. An environmental mismatch can occur when prenatal and postnatal environments lack compatibility, which then could increase the individual’s susceptibility to adult diseases [39]. Prenatal and postnatal nutritional factors also play an important part in brain development which can track into later life [17, 40, 41]. As nutrients influence brain development, the roles of maternal diet during pregnancy, milk feeding and complementary feeding in cognitive development require further investigation. Understanding the influence of variations in maternal and infant nutrition on growth and cognitive development in the first 2 years of life is essential for developing strategies to improve overall growth and development of children as well as to lower the risk of adult diseases.

In Malaysia, despite rapid economic growth and development and improvements in socio-economic status and health care system, child under-nutrition still persists particularly in low-income communities. The prevalence of underweight and stunting among children aged below 18 years was 13.2 % and 17.2 % in 2006 [42], while in 2011, 16.1 % and 13.4 % of the children were found to be underweight and stunted respectively [43]. In 2015, the prevalence of underweight and stunting among Malaysian children was 13.0 % and 13.4 % respectively [44]. At the same time, childhood obesity is also increasing in Malaysia. The prevalence of obesity among Malaysian children below 18 years old has increased from 6.1 % in 2011 [43] to 11.9 % in 2015 [44]. In another nationwide survey on nutritional status of children aged 6 months to 12 years, the overall prevalence of overweight and obesity among the children was 9.8 % and 11.8 % respectively [45].

Overweight and obesity are risk factors for non-communicable diseases (NCDs). In Malaysia, the prevalence of overweight and obesity among Malaysian adults have increased from 16.6 % and 4.4 % respectively in 1996 [46] to 29.1 % and 14.0 % respectively in 2006 [47]. The prevalence further increased to 29.4 % and 15.1 % respectively in 2011 [43] and to 30.0 % and 17.7 % respectively in the recent National Health and Morbidity Survey (NHMS) in 2015 [44]. Hypertension and hypercholesterolemia, which are risk factors for cardiovascular diseases (CVDs), are also prevalent among Malaysians. For adults aged 18 years and above, the prevalence of hypertension has increased from 29.9 % in 1996 [46] to 32.2 % in 2006 [47] and 32.7 % in 2011 [43]. However, in 2015, the prevalence has slightly decreased to 30.3 % [44]. For hypercholesterolemia, the prevalence shows an increasing trend with 11.5 % in 1996 [46], 20.7 % in 2006 [47], 32.6 % in 2011 [43] and 47.7 % in 2015 [44]. Over the years, Malaysia has also seen increasing prevalence of diabetes mellitus in adults 18 years and above with 11.6 % in 2006 [47], 15.2 % in 2011 [43] and 17.5 % in 2015 [44].

The rising prevalence of non-communicable diseases among adults in Malaysia supports the need to investigate pre-natal and post-natal factors that could be related to the observed disease trends in Malaysia. The USM Pregnancy Cohort Study, which was conducted on 153 Malay women in Kelantan in 2009, was the first study that examined the concept of early nutritional programming [48]. The study reported that higher intakes of fruits and vegetables during pregnancy were associated with higher birth weight, birth length and head circumference at birth [49]. In addition, higher maternal pre-pregnancy BMI was associated with higher BMI-for-age and weight-for-age of infants at 12 months of age [50]. The present study which aims to recruit a larger number of mother and infant pairs will be able to complement the USM Pregnancy Cohort Study on providing insights into the link between early nutrition with growth and cognitive development of infants in Malaysia.

## Conclusion

Data from this study will contribute to the gap in knowledge related to growth and cognitive development during the first 2 years of life. This study is expected to demonstrate an association between early life nutrition with growth and cognitive development of infants. The findings of this study can be used to develop evidence-based recommendations on infant nutrition related to growth and cognitive development. Any associated effects of early nutrition on growth and cognitive development may provide a basis for development of relevant intervention strategies as well as strengthening existing strategies and policies related to maternal and child health in Malaysia.

## Abbreviations

BMI: Body mass index
BSID-III: Bayley Scales of Infant and Toddler Development, Third Edition
CVDs: Cardiovascular diseases
ECD: Early Childhood Development
EDPS: Edinburgh Post Natal Depression Scale
FAO: Food and Agriculture Organization of the United Nation
IBQ-R: Revised Infant Behavior Questionnaire
IDA: Iron Deficiency Anemia
IPH: Institute for Public Health
IT-HOME: Infant Toddler HOME Inventory
IYCF: Indicators for assessing infant and young child feeding practices
JKEUPM: Universiti Putra Malaysia Ethics Committee for Research Involving Human Subjects
MUAC: Mid-upper arm circumference
NCDs: Non-communicable diseases
NHMS: National Health and Morbidity Survey
S-FFQ: Semi-quantitative food frequency questionnaire
SPM: Standard Progressive Matrices
SSF: Subscapular skinfold thickness
TSF: Tricep skinfold thickness
USDA: United States Department of Agriculture
USM: Universiti Sains Malaysia
WHO: World Health Organization
